# Polymer Flexible Joint as a Repair Method of Concrete Elements: Flexural Testing and Numerical Analysis

**DOI:** 10.3390/ma13245732

**Published:** 2020-12-16

**Authors:** Łukasz Zdanowicz, Szymon Seręga, Marcin Tekieli, Arkadiusz Kwiecień

**Affiliations:** Faculty of Civil Engineering, Cracow University of Technology, 31-155 Cracow, Poland; sserega@pk.edu.pl (S.S.); mtekieli@pk.edu.pl (M.T.); akwiecie@pk.edu.pl (A.K.)

**Keywords:** polyurethane, flexible joint, concrete repair, stress redistribution

## Abstract

Polymer Flexible Joint (PFJ) is a method for repairs of concrete elements, which enables carrying loads and large deformations effectively. This article presents the possibility of applying PFJ on beams subjected to bending and describes the influence of such joints on concrete elements. An experimental investigation was conducted to determine the behavior of concrete in a four-point bending test. The research program included flexural tests of plain concrete elements with a notch, as well as tests of elements which were repaired with PFJ after failure. Based on the experimental results, the numerical characteristics of analyzed polymer and concrete were calibrated. A nonlinear numerical model is developed, which describes the behavior of concrete elements and polymer in the experiments. The model is used to numerically analyze deformations and stresses under increasing load. The influence of flexible joint on concrete elements is described and behavior of elements repaired with PFJ is compared to original elements. Particular attention was paid to the stress redistribution in concrete. The application of flexible joint positively influences load capacity of the connected concrete elements. Furthermore, because of stress redistribution, connected elements can bear larger deformations than original ones. PFJ can therefore be considered an efficient repair method for connecting concrete elements.

## 1. Introduction

### 1.1. Motivation

**Figure 1:**
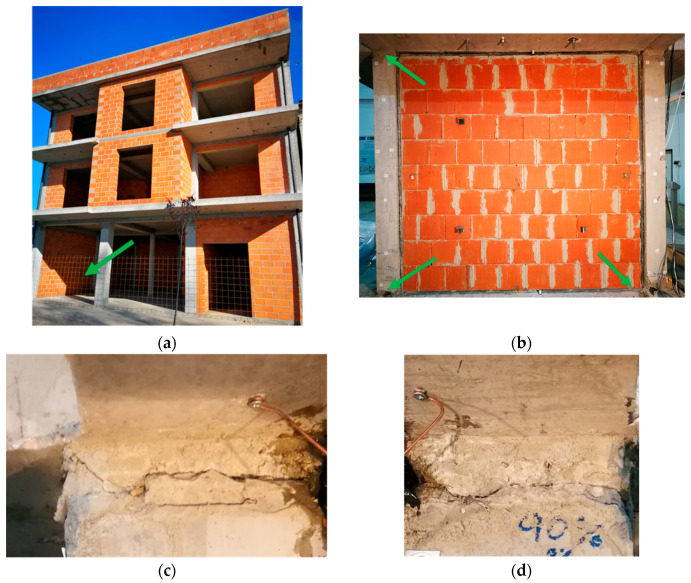
RC frame building with infill walls (**a**), infill specimen [3] excited on shake table with columns damaged at top and bottom (**b**), damages (hinges) of concrete in RC column tops (**c**,**d**).

Early-age shrinkage as well as mechanical cracks in concrete are common problems in constructions. Deformations caused by concrete shrinkage or temperature changes leads to local stress concentrations in the concrete. This results in damage of concrete components such as floors or roadways [1,2]. In addition, mechanical actions cause damage to concrete used in structural elements by overloading them. Earthquakes are the most serious excitations, resulting in concrete cracking and plastic, e.g., in infill structures (Figure 1)—visible for a specimen tested on shake table [3]. Cracked concrete is unable to transfer tensile forces thus various repair and strengthening methods of it are used [4]. Filling of cracks by external injection with bonding material such as epoxy resin or self-healing methods [5,6] use mainly stiff and high strength, but hardly deformable materials, which generate stress concentrations in concrete weakened by micro-cracks [4]. Recovering of strength and stiffness in damaged cross-sections leads also to the use of composite materials [7,8] with various adhesives [9,10].

**Figure 2:**
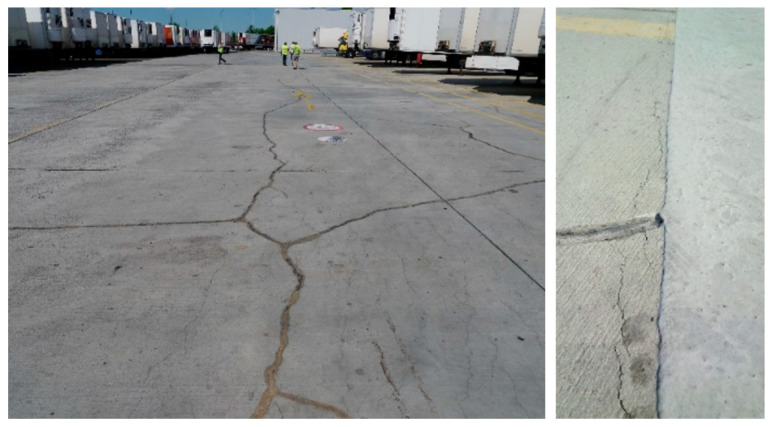
Damage examples: concrete pavement with cracks caused by shrinkage (**left**) and after repair with epoxy resin (**right**).

Common joint materials, such as epoxy resins, have high stiffness and low deformability, which can be a disadvantage, since new cracks often appear after repair, close to the repaired one. On the contrary, sealants or mortars which are also used for such repairs do not have sufficient strength to carry internal forces (Figure 2) [11,12,13]. A good solution is to adjust the stiffness and strength of the joint material so that it is suitable for use in certain situations. This is also important considering the sustainability of buildings and Sustainable Development Goals strategy [14,15]. One option that meets these requirements is the Polymer Flexible Joint (PFJ) method.

### 1.2. Idea of the Polymer Flexible Joint

Polymer flexible joint is a method that allows effectively bearing the loads and absorbing large deformations at the same time [16]. Therefore, it can be suitable for repairing damaged structures (mostly fragile building materials) as well as for joining new concrete elements. PFJ is based on polyurethane, which is a material with highly effective bonding and hyper-elastic properties. Several types of PFJ are available, with tensile strength ranging from 1.7 to 20 MPa, and elongation at rupture between 10% and 120%. Currently, PFJ research is focused on the application of PM-type, PS-type, and PT-type polyurethanes, which can be used as injection or prefabricated elements, named also as PUFJ (PolyUrethane Flexible Joints) [3,17,18,19]. The PM-type has the lowest and the PT-type the highest values of stiffness and strength, while the properties of the PS-type are between the other two types.

This article presents experimental and numerical investigations of the most effective PT-type polymer for concrete repair. It can be applied in cracks of concrete and RC elements making elastic (or nonlinear elastic) hinge, which is able to transfer bending loads and high deformation simultaneously, also under cyclic loads. It can be used for the repair of cracked concrete surfaces (Figure 2). Moreover, damages to columns after earthquakes (forming plastic hinges) requires a quick emergency repair before an aftershock. Because of the short time between the main shock and the aftershock (hours, days), a PT-type polymer can be used as a temporary repair injection and confinement, replacing crushed concrete (Figure 1c,d) and making visco-elastic hinge (with remained steel reinforcement), withstanding dynamic loads and introducing in the structure ductile and damping properties.

### 1.3. Research Aim

The aim of the research presented in this article is to investigate the possibility of the application of PFJ on beams subjected to bending. Until now, PT-type polymer was not thoroughly investigated in flexure. The four main objectives of this work are: (1) to describe the influence of PFJ on concrete elements and stress redistribution, (2) to determine the behavior of concrete in four-point bending tests, including digital image correlation analysis, (3) to perform a numerical analysis of the experimental work, describe the behavior of concrete elements and polymer and analyze deformations and stresses under increasing load, and (4) to assess the influence of flexible joint on concrete elements and compare the behavior of elements repaired with PFJ to original elements, focusing on joint effectiveness.

## 2. Experimental Research

### 2.1. Materials

CONCRETE. All concrete specimens were made of one concrete mixture: normal concrete based on Portland cement with a *w/c* ratio of 0.45 and a maximum aggregate size of 16 mm. To determine the compressive strength of the concrete, 150 mm cubes were used, while the tensile strength was measured in the uniaxial tensile test on prisms with dimensions of *b* × *h* × *L* = 100 × 100 × 200 mm^3^ with a notch *a* = 25 mm on both sides in the center of each prism. These tests were carried out 60 days after casting [20]. It can be classified as C 45/55 class.

POLYMER. A two-component PT-type polymer was used in the research program. It is an elasto-visco-plastic material with good bonding properties. The tensile test was carried out according to EN ISO 527-1 [21] at the strain ratio of 1%/min [18]. The compressive tests in accordance with ISO 7743 were conducted [22]. The Poisson ratio of the PT-type polymer is 0.495 [16]. Young modulus of PT-type polymer is *E* = 700 MPa, almost two orders lower than the applied concrete [18]. Table 1 shows the mechanical properties of concrete and polymer.

### 2.2. Testing Methodology

The experimental investigations consist of 63 four-point bending tests (4PB) with various structural materials. The test specimens were divided into 21 series, each containing 3 test specimens. The aim of this research program was to analyze the influence of the polymer PT on joint effectiveness (JE) [23,24] in concrete crack repair for one chosen series. The results of other series are not a subject of this article.

Joint effectiveness was defined as a ratio of load-bearing capacity or elongation capacity of the elements after the repair and before the repair. In this paper, experimental and numerical analysis of one series (3 test specimens before and after repair) for concrete and polymer PT cooperation is presented.

**Figure 3:**
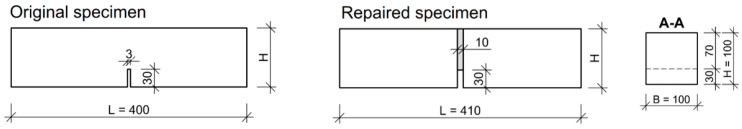
Specimen geometry (dimensions in mm): original specimen and specimen after repair (the roughness of the repaired concrete surface at the polymer-concrete interface is idealized in the presented scheme).

The specimens used in 4PB tests were prismatic, with a length of *L* = 400 mm and a square cross-section with *b* × *h* = 100 × 100 mm^2^. Each specimen was provided with a notch of *a* = 30 mm depth at the bottom, in the middle of its length (see Figure 3). Such elements were called original elements (elements without PFJ). They were tested for bending and then repaired with a PT-type polymer after failure. The repair was carried out with a joint thickness of *t* = 10 mm, to simulate large crack with and loss of crushed concrete surface. The elements after the repair are later referred to as “repaired elements”. Before the repair, the concrete surface was cleaned and a layer of primer (SIKA ZP Primer—a single component, ready to use, chemically hardening, containing solvents polyurethane primer, Sika Poland, Cracow, Poland) was applied. The bending tests of the repaired elements were carried out 16–20 h after the polymer application (a kind of emergency repair).

The bending tests were performed with a Zwick/Roell Z100 testing machine (Ulm, Germany). The test parameters are as follows:

- Maximum force:     100 kN,
- Initial force:       0.80 kN,
- Test displacement rate:  0.10 mm/min,
- Span length:       300 mm,
- Distance between loads: 100 mm.

**Figure 4:**
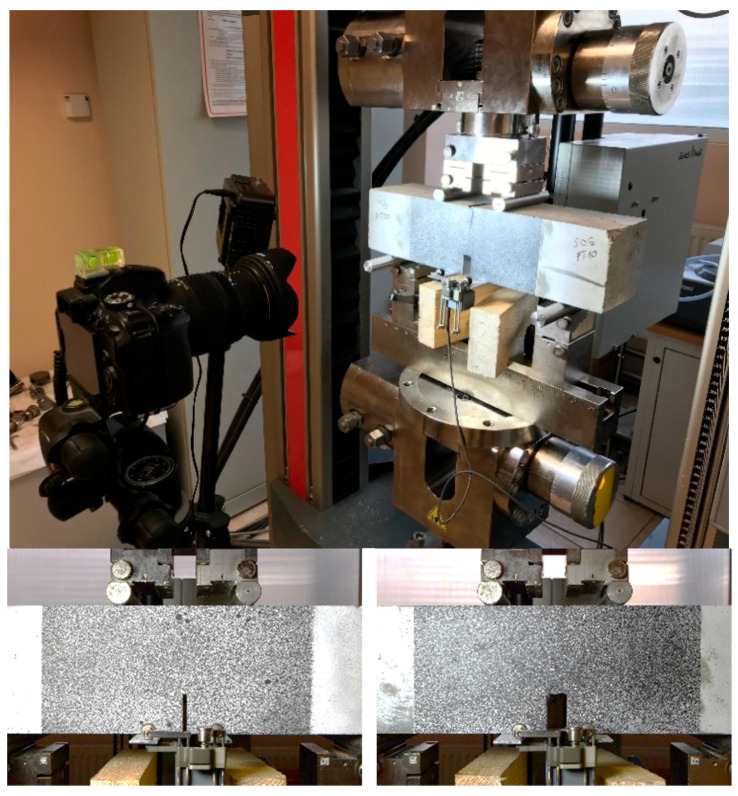
Test setup for four-point bending test (**top**) with DIC field of the original specimen (**bottom left**) and the repaired specimen (**bottom right**).

Three measurement methods were used simultaneously in all 4PB tests: (1) extensometer for the crack width displacement (CMOD), (2) extensometer for the vertical deformation, (3) digital image correlation (DIC) to measure the strain field on the side surface of the specimens (Figure 4). For DIC method the CivEng Vision system (Cracow, Poland) was used [25,26].

### 2.3. Test Results

#### 2.3.1. Load Response

**Figure 5:**
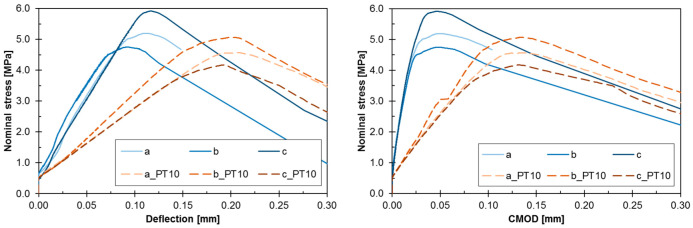
Load response in terms of stress-deflection (**left**) and stress-CMOD (**right**) of the notched beam in 4PB test (continuous lines for original elements, dashed lines for repaired ones).

Figure 5 shows the stress-strain relationship in bending tests for specimen-series *b* × *h* = 100 × 100 mm^2^. The curve is linear up to the maximum load for both original and repaired elements. It is noted that repaired specimens, where the polymer was used, have a lower bending stiffness than original specimens. Therefore, higher CMOD values were observed for repaired specimens at the same load levels. The load-bearing capacity of original elements reached higher values in most cases. Before maximum nominal stress σ*_max_* (in the pre-critical phase where σ*_max_* = 6 × *M_max_*/(*b* × *h*′^2^)), no cracks were detected with the unarmed eye. However, crack formation was observed in the post-critical phase (≥σ*_max_*).

#### 2.3.2. Failure Mode

**Figure 6:**
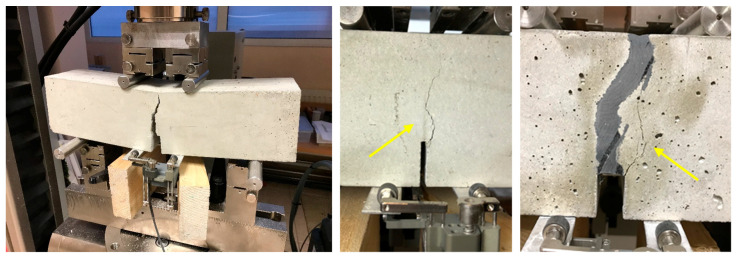
Failure of the original specimen (**left**) and main crack in original (**middle**) and repaired specimen (**right**).

**Figure 7:**
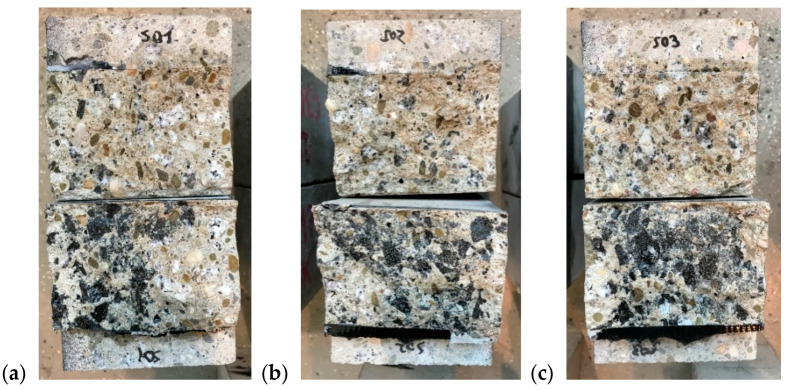
Failure surface of the repaired specimens: (**a**) specimen S01, (**b**) S02, (**c**) S03.

All specimens had a similar type of failure—a crack occurred along the notch where the stresses reached the highest values. Furthermore, the failure was sudden and brittle. Cracks run not only through the cementitious matrix but also through aggregates (Figure 6 and Figure 7). In the repaired specimens, the failure was also caused in form of crack in the concrete and the crack developed 1–3 mm from the notch. No specimen failed due to damage through the polymer in the joint.

#### 2.3.3. Digital Image Correlation Results

**Figure 8:**
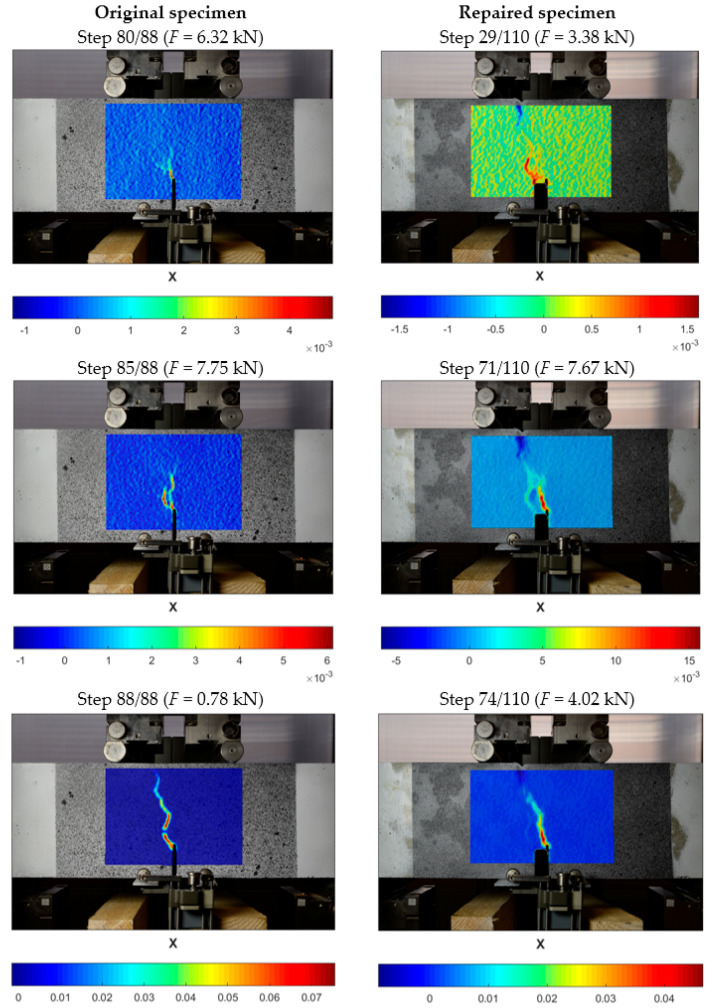
Crack development under applied load for original specimen (**left**) and repaired specimen with PT-type polymer joint of 10 mm (**right**); results obtained using CivEng Vision software.

Using digital image correlation method (DIC), first cracks were already observed before reaching the maximum load. A development of the horizontal strains (X-axis) of the original and repaired specimens is shown in Figure 8. In the original specimens, first cracks were visible on the left side of the notch a few steps before failure (load step 80/88). These cracks developed further and joined to form a main crack when the failure occurred (load step 88/88). The main crack followed the shortest path through the cross-section and no further cracks were visible in the damage area.

Repaired specimens had a different mechanism. The first cracks appeared much earlier, which was a consequence of the already weakened cross-section (during the previous test of the original specimen). In the repaired specimen several main cracks developed further (load step 29/110) on the left side of the notch. However, halfway through the test a new crack developed on the other side of the notch (load step 71/110). From this point on, the previous, initial cracks did not develop further, and the new crack caused the failure. This sidewise shift of the damage process is visible between the last steps (load step 68/110 and 71/110). The horizontal strain at *F_max_* of the repaired specimens was about 2.5-times higher than of the original specimens.

The phenomenon above confirms observation presented in [4] for tensile tests of concrete specimens repaired with PT-type polymer that the PFJ can cause mechanical closing of micro-cracks in concrete by redistribution of stress and protect the weakened (by micro-cracks) concrete zone against damage in the same place. In the analyzed case, stress redistributed by the PFJ in the failure cross-section found another place with higher level of fracture energy, where new damage occurred.

#### 2.3.4. Joint Effectiveness

As mentioned above, the load-bearing capacity of the repaired elements was generally lower than the original ones. The average values of maximum stress for original and repaired elements are 5.28 MPa (SD = 0.59 MPa, CV = 11.1%) and 4.60 MPa (SD = 0.45 MPa, CV = 9.8%) respectively. On the other hand, the average values of CMOD are 47.7 μm (SD = 0.07 μm, CV = 1.5%) and 132.7 μm (SD = 1.1 μm, CV = 0.8%) respectively. The repaired specimens manifested load bearing capacity of 87% of the original elements, but ductile behavior increased almost three times.

**Figure 9:**
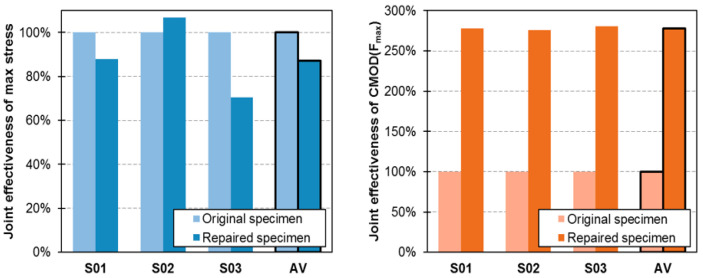
Joint effectiveness of polymer flexible joint with PT-type polymer in terms of maximal stresses (**left**) and CMOD at maximal load (**right**).

A comparison of maximum stresses and maximum CMODs for the original and repaired specimens is shown in Figure 9. For each test specimen series, the maximum of average stresses and the maximum of average CMOD values are presented graphically and the effectiveness of the repair in terms of force and CMOD is calculated.

In terms of maximum stress, the repaired specimens achieved only 13% lower strength than the original specimens. In terms of deformability, the repaired specimens achieved significantly higher deformation at break than the original ones. This is explained by the substantially higher deformability of polymer of the hyperelastic characteristic when compared to brittle concrete. In general, the specimens repaired with a 10 mm polymer layer had the average CMOD value 2.78 times higher than the specimens before the repair. These two joint effectiveness values indicate increase in global fracture energy of the specimens after repair, what is not observed when brittle materials (cracked) are repaired (bonded) using stiff and high strength materials [27]. Typical epoxy resins are of about two orders higher stiffness (*E* = 30,000 ÷ 40,000 MPa) than the applied PT-type polymer (*E* = 700 MPa). On the other hand, ability of PT-type polymer to deform up to 10% of ultimate strain with hyperelastic characteristic results in stress redistribution, thus the PFJ can act as a load-bearing connection and absorb large deformations at the same time.

## 3. Numerical Analysis

### 3.1. Finite Element Model

**Figure 10:**
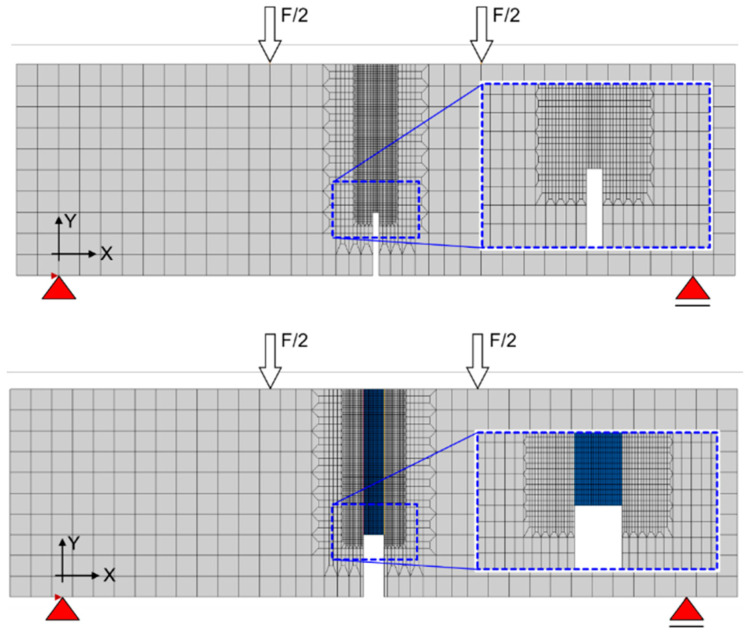
Finite element model for original specimen (**top**) and repaired specimen (**bottom**).

The numerical analysis of the 4PB test was performed with the DIANA Finite Element Software [28] in 2D as a plane stress state. The topology of a FE-mesh used in the analysis is shown in Figure 10. The mesh consists of square isoparametric plane stress elements with square shape function (type *CQ16M*) of eight nodes [28]. The maximum size of a single element is 10 mm and the minimum size is 1 mm (elements in the notch area). To ensure the correct behavior of the concrete-polymer contact zone, additional contact elements (type *CL12I*) with zero thickness at the contact area with 95% tensile strength of the concrete were used. Boundary conditions were defined as rigid supports. The right support was modelled as a sliding type in the horizontal direction without taking any possible friction effect.

### 3.2. Constitutive Material Models

The constitutive model for concrete used in the analysis is based on a smeared crack model [29] and was formulated in total strains according to the concept of [30,31] and DIANA algorithm [28]. A rotating crack model is used in the analysis. A linear-elastic stress-strain relationship is assumed for concrete in compression. The concrete tensile behavior is described by exponential post-cracking behavior in the following form [28]:

(1) $$\sigma_{nn}^{cr}\left(\varepsilon_{nn}^{cr}\right)=exp\left(-\frac{\varepsilon_{nn}^{cr}}{\varepsilon_{nn.ult}^{cr}}\right)$$

(2) $$\varepsilon_{nn.ult}^{cr}=\frac{G_{f}}{h\cdot f_{t}}$$

where $\sigma_{nn}^{cr}$ is the stress perpendicular to the crack, $\varepsilon_{nn}^{cr}$ is the strain in the same direction and $G_{f}$ is the fracture energy, $f_{t}$ is the tensile strength of the concrete and *h* is a crack bandwidth, which is assumed here as the square root of a finite element area. Table 2 shows the material constants used for concrete and polymer in the numerical simulations.

The maximum tensile stresses in the system were equal to the concrete tensile strength; in this range, the polymer behaved as a linear-elastic material [18]. Therefore, the polymer was modelled as linear elastic (*f_ct_* << *f_pt_*).

**Figure 11:**
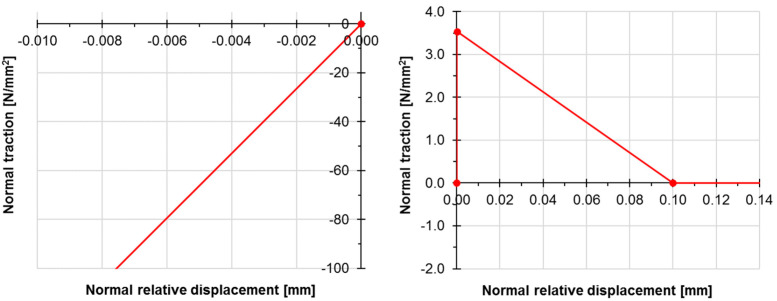
Mechanical parameters of interface between concrete and polymer for compression (**left**) and tension (**right**).

To take this into account, the influence of the primer layer between the damaged concrete and the polymer, a zero thickness interface layer was modelled. A non-linear model of the interface was adopted with a strength equal to 95% of the concrete tensile strength. The remaining parameters were calibrated empirically on the basis of experimental results (Figure 11).

### 3.3. Solution Strategy

An incremental-iterative solution approach according to the Newton–Raphson method was used. For the first steps of analysis (up to load *F* = 6000 N) a load control procedure was carried out; the load step in this phase was equal to 200 N. After reaching load *F* = 6000 N and before the cracking load, the system switched to an arc-length control procedure with load step of 100 N. Convergence criteria assumed the force and displacement norm. Due to the relatively small deformations, the analysis only took into account material non-linearity, while non-linear geometrical analysis was excluded. Two master control nodes were located at the lower edge of the specimens next to the notch. The results of these nodes shift were called CMOD deformation.

### 3.4. Numerical Results

The main objective of the analysis was to compare the developed numerical model with experimental results. The values of the maximal forces from experiment differ in comparison to the values from the numerical analysis by less than 9% for the original specimens and less than 7% for the repaired specimens (Table 3). The joint effectiveness obtained from the experimental tests was thus 87% and from the numerical calculation 88%.

In relation to the CMOD at maximal load (cracking load), the difference between experimental results and calculation was 43% for the original test specimens and 1% for the repaired specimens. Such a high difference in deformation results was due to the stiffness of the test bench. It should be noted that the difference of the CMOD at cracking load between original and repaired specimen in the numerical analysis was 4.8 times.

**Figure 12:**
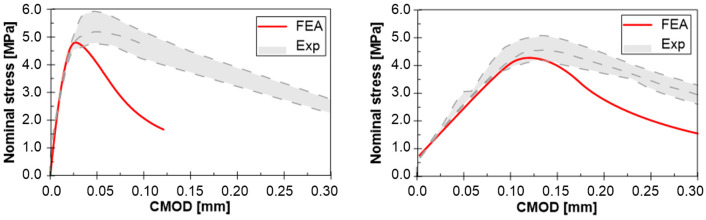
FE vs. experiment results—Nominal stress-CMOD diagram for original (**left**) and repaired specimen (**right**).

Figure 12 shows the experimental and numerical stress-strain (CMOD) relationship. The numerical analysis of the original specimens correctly reproduced the path in the pre-critical phase. However, the difference is clearly visible in the post-critical phase, where experimental specimens behave more ductile. Possible explanation is the arc failure mechanism due to friction at both supports. Such phenomenon was not modelled in the FEM. Friction on supports increases the load-bearing capacity of bending elements due to the change in the direction of main stress. In fact, the main compressive stresses σ_1_ ran along the curve line (vault effect) and near the supports their direction was not parallel to the edge of the element. It should be emphasized, however, that the numerical model correctly reproduces the pre-critical phase and failure mechanism.

Similar to the original specimens, the behavior of the repaired specimens in the FE-analysis is comparable to the experimental results. It is pointed out that not only the pre-critical path was reproduced correctly, but also for the post-critical phase the numerical results are satisfactory. Probably, using of hyperelastic model for the polymeric material (instead of the linear one) [9] can predict better the post-failure path.

Comparison of initial (original specimen) and subsequent (repaired specimen) damage energy, evaluated by the area under curves in Figure 12, also confirms this observation (damage energy is defined here as an area under stress-CMOD curve up to the maximum load). The average damage energy obtained in experiment was equal to 0.193 N/mm and 0.408 N/mm for original and repaired specimens, respectively. Even if the ultimate stress was lower in the cases of repaired specimens, more ductile behavior of the specimens after repair using PFJs was advantageous.

**Figure 13:**
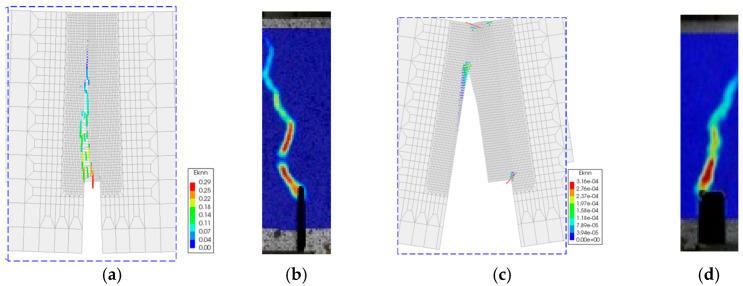
Failure mode and crack strains at failure in X-direction of original specimen—FE-calculation at 50× magnification (**a**), experiment acc. to DIC map (**b**) and repaired specimen—FE-calculation at 50× magnification (**c**), experiment acc. to DIC map (**d**).

The numerical model could also predict the crack formation (Figure 13). The specimens were damaged by cracks forming along the notch where the tensile stresses reached their highest value. It can be observed that the path of the crack pattern is similar to the strain results from DIC.

## 4. Summary and Conclusions

The experimental results of the four-point bending tests on plain concrete beams and beams repaired with PFJ specimens were presented together with the numerical analysis based on FE-modelling. The following conclusions can be drawn from a comparative analysis of the experimental and numerical results:

(1) The joint effectiveness of Polymer Flexible Joint with PT-type polymer in terms of load-bearing capacity is 87% (on average) in the experiments and 88% in the numerical analysis.
(2) For specimens repaired with PT-type polymer, the polymer is decisive for the experimental and numerical results in terms of CMOD values. It was observed that the strain capacity of the repaired specimens was more than 280% higher than that of the original specimens.
(3) The repaired specimens were able to manifest higher damage energy than the original ones; the area under stress-CMOD curve for repaired specimens was more than 2× higher than that of the original ones.
(4) It is emphasized that the specimens repaired with PFJ have the load-bearing capacity of more than 80% of the original ones. In this way the PFJ can be used as a load-bearing connection. In addition, the higher flexibility of the connection leads to a reduction of possible imposed stresses and introduces additional ductile behavior of structural elements after repair.
(5) Using DIC method, the phenomenon of stress redistribution around a flexible joint was documented.
(6) The comparison between experimental and numerical analysis shows a good agreement of the results in terms of failure load and deformation (CMOD values).
(7) The numerical model correctly reproduces the pre-critical phase and failure mechanism. It was proven that presented numerical simulations can be a useful and suitable tool for the analysis of pre-critical phase of a four-point bending test of concrete bonded with a polymer flexible compound.
(8) However, the post-critical path was not satisfactory (lower stiffness in the results from numerical calculations than from experiments). Experimental results show much smoother curves during failure and in the post-critical phase than numerical calculations. The reason for this may be the friction of the support during the test, where a quasi-arch effect might have occurred. Further investigations are required to adequately describe the mechanism in the post-critical phase.

## Figures and Tables

**Table 1:** Concrete and polymer mechanical properties [18,22,23].

Test	AV ^1^ [MPa]	SD ^2^ [MPa]	CV ^3^ [%]
Concrete compressive strength, *f_cm_*	68.9	3.57	5.2
Concrete tensile strength, *f_ctm_*	3.73	0.34	9.1
Polymer PT-type compressive strength, *f_pc_*	26.8	1.29	4.8
Polymer PT-type tensile strength, *f_pt_*	18.8	1.36	7.2

^1^ AV—average value; ^2^ SD—standard deviation, ^3^ CV—coefficient of variation.

**Table 2:** Mechanical properties of concrete and polymer.

Material	*E* [MPa]	ν [-]	*f_t_* [MPa]	*G_f_* [N/mm]
Concrete	36,700	0.20	3.73	0.150 ^1^
Polymer PT-type	700	0.49	20.0	n/a

^1^*G_f_* = 73 *f_cm_*^0.18^ = 0.150 N/mm [32].

**Table 3:** Results comparison of experimental tests and numerical calculations.

Parameter	Original Specimen		Repaired Specimen	
	Experiment	FE-Analysis	Experiment	FE-Analysis
Failure load, *F_max_* [kN]	8.63	7.84	7.52	7.00
CMOD at *F_max_* [μm]	47.7	27.4	132.7	131.2
Strain at *F_max_*, *E_knn_* [-]	0.071 ^1^	0.038	0.015 ^1^	0.005
Damage energy [N/mm]	0.193	0.087	0.408	0.318

^1^ value obtained from the results of the DIC analysis.
